# Recognizing Physisorption and Chemisorption in Carbon Nanotubes Gas Sensors by Double Exponential Fitting of the Response

**DOI:** 10.3390/s16050731

**Published:** 2016-05-19

**Authors:** Andrea Calvi, Alberto Ferrari, Luca Sbuelz, Andrea Goldoni, Silvio Modesti

**Affiliations:** 1 Department of Physics, University of Trieste, via A. Valerio 2, 34127 Trieste, Italy; andrea.calvi@studenti.units.it (A.C.); alberto.ferrari@studenti.units.it (A.F.); luca.sbuelz@studenti.units.it (L.S.); Silvio.Modesti@ts.infn.it (S.M.); 2 Elettra-Sincrotrone Trieste, S.S. 14, km 163.5, 34149 Trieste, Italy; 3 CNR-IOM TASC, S.S. 14, km 163.5, 34149 Trieste, Italy

**Keywords:** CNTs sensors, chemisorption, physisorption

## Abstract

Multi-walled carbon nanotubes (CNTs) have been grown *in situ* on a ${\mathrm{SiO}}_{2}$ substrate and used as gas sensors. For this purpose, the voltage response of the CNTs as a function of time has been used to detect $H_{2}$ and ${\mathrm{CO}}_{2}$ at various concentrations by supplying a constant current to the system. The analysis of both adsorptions and desorptions curves has revealed two different exponential behaviours for each curve. The study of the characteristic times, obtained from the fitting of the data, has allowed us to identify separately chemisorption and physisorption processes on the CNTs.

## 1. Introduction

Resistive-type gas sensors are devices that detect a change in the concentration of a chemical specimen through the variation of an electric signal. The main features of good gas sensors are high sensitivity and selectivity, fast response, low cost production and high reliability [1,2]. Gas sensors are often used for public safety (e.g., to detect explosive or toxic leaks), in environmental and food monitoring, in the pharmaceutical industry, in clinical diagnostic and in medical engineering [3,4].

Nanostructured materials have attracted considerable interest as gas sensing components: their high surface/volume ratio increases the adsorption of gases and therefore enhances their sensitivity to adsorbates. Besides, nanodevices are very compact objects and they usually dissipate little power in current transport [5]. In the past two decades, many studies were performed on the sensing properties of semiconducting oxides such as ZnO, ${\mathrm{SnO}}_{2}$ and ${\mathrm{In}}_{2}O_{3}$. However, those sensors showed issues regarding the long-term stability and the capability to operate at room temperature [6]. Recently, a large effort has been put into developing carbon nanotubes (CNTs [7,8]) based sensors. These sensors are very promising since they are characterized by short response and recovery times, high sensitivity and stability and wide temperature operating range [5].

Gas sensing properties of CNTs can be related to a change in some physical properties induced by the absorption of molecules, namely a change in conductivity, resistivity, dielectric constant, *etc.* [9,10,11,12,13,14,15,16,17,18,19,20,21]. In this work the sensing properties of CNTs are studied through the variation of the resistivity.

The adsorption can take place with two different processes, namely, chemisorption and physisorption. While the former process is related to the bond formation between the adsorbate and the adsorbant, the latter is characterized by dipole and Van der Waals interactions between the adsorbate and the adsorber. The two processes are easily distinguishable in most cases due to the differences both in the energy scale as well as in the desorption time scale, opposing a generally faster physi(de)sorption to a slower chemi(de)sorption. Both chemisorption and physisorption can induce a change in resistivity of CNTs sensors. This variation is due to a net charge transfer between molecules and the CNTs array in the former case and to a change in the electron (and hole) carrier mobility in the latter.

Resistivity (as well as conductivity) response of CNTs sensors has been widely studied [22] in the last decade, due to the advantages related to nano-structured sensors array. For both CNTs and graphene negative and positive resistivity variations have been observed, depending on the electronic properties of the gas exposed to the sensors [23,24,25]. A lot of effort has been made in characterizing the response of CNTs sensors to toxical gases, e.g., nitrate compounds [26,27]. Many experiments have focused on the capability to reach good chemical selectivity studying the characteristic curves of the process [11,26]. Although some quantitative analysis has been carried out, little has been done to characterize the adsorption process taking place, whether chemisorption, physisorption or both [28]. From the theoretical point of view, however, the adsorption process on CNTs is well understood: first principle calculations for nitrogen dioxide (${\mathrm{NO}}_{2}$), ammonia (${\mathrm{NH}}_{3}$) [12] and hydrogen ($H_{2}$) [29] on CNTs have shown that both chemisorption and physisoprtion are present and furthermore the former can take place in two different sites:

- on the surface of a nanotube (mainly on defects),
- inside CNTs bundles.

Since the theoretical calculations suggest that both chemisorption and physisorption are active processes, but still doubts remain from the experimental point of view [27], the aim of this paper is to understand whether or not it is possible to recognize the kind of adsorption process from the analysis of the resistivity response induced by the gas. The gases used for this task are $H_{2}$ and ${\mathrm{CO}}_{2}$, which are both known to induce a positive variation of resistivity for CNTs arrays [14,16,17,18,30].

## 2. Experimental

A total of four CNTs samples were prepared using the chemical vapor deposition (CVD) technique [31,32], by evaporating gaseous acetylene ($C_{2}H_{2}$) over a silicon oxide substrate (approximate dimensions: 1.5×2×0.05 cm), covered with iron nano-particles. The samples were put in HV condition at a pressure of $10^{-6}$ mbar and annealed to a temperature of $650^{\circ }$C. At that constant temperature, they were treated with hydrogen ($H_{2}$) at a static pressure of $10^{-1}$ mbar for 10 min. After the recovery of the initial vacuum pressure, a subsequent exposure of $C_{2}H_{2}$ at a dynamic pressure of $10^{1}$ mbar for 10 min was performed. The samples were then gradually cooled down to room temperature and collected. Finally, two sides of the samples were painted with a silver-flakes paint, in order to create two electrical junctions for measuring the resistivity response.

**Figure 1:**
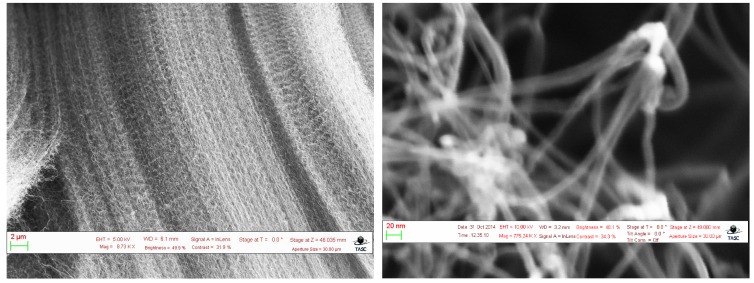
(**Left**) A Scanning Electron Microscope (SEM) image from the vertically aligned grown samples; (**Right**) Particular of the tips from the grown nanotubes. From this image it is clear that the natotubes twine at the tips. The diameter of the carbon nanotubes (CNTs) can be estimated to be around 10 nm.

The samples were observed through a Scanning Electron Microscope (SEM) (Figure 1). Although the arrangement of the CNTs showed a mostly vertical alignment, the CNTs were observed to join in bundles of 5–10 tubes. From the SEM images the diameter of the tubes was estimated to be around 10 nm, implying that multi-walled CNTs were grown.

The variations of the electrical resistivity of CNTs were investigated by measuring the voltage across the sample as a function of time, keeping a constant current of 5 mA. The same measurements can be performed with constant voltage bias, but no advantage is found in terms of overall accuracy. Indeed, the CNTs were electrically connected to a Keithley 2400, which was used both as a current generator and as a measurement device. The data acquisition was made possible by interfacing the Keithley with a Labview program, that returned curves of voltage as a function of time. For measuring the response due to gas exposure, the samples were put in HV conditions. A thermocouple was needed in order to perform measurements at constant temperature. To measure the pressure inside the experimental chamber, an active ion gauge was used in the range $10^{-2}$–$10^{-6}$ mbar.

The voltage across the CNTs was measured with a sampling of 500 ms. The acquiring system was started and background data were collected for 5 min, in order to obtain a voltage offset. A gas was then inserted in the chamber via a leak valve, and the response of the system was measured, if any. After an interval of time ranging from 5 to 10 min after the gas injection, the valve was closed and the gas was pumped out. Before starting a new cycle, the system was left recovering to a new equilibrium voltage.

## 3. Results and Discussion

**Figure 2:**
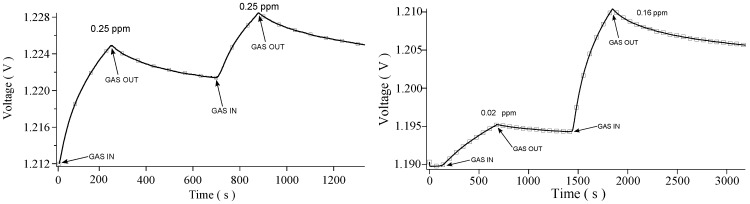
Typical set of voltage vs time data. The response can be also seen as variations in measured resistivity. Measured data for the hydrogen case. (**Left**) Exposure to 0.25 ppm; (**Right**) Exposure to 0.02 and 0.16 ppm.

Data have been collected for two different species: hydrogen ($H_{2}$) and carbon dioxide (${\mathrm{CO}}_{2}$). In the following, a total of five and two sensing cycles are respectively analyzed; every cycle is characterized by exposing the sensor to a certain gas concentration. A typical response signal consists in a set of points of measured voltage across the sensor as a function of time as shown in Figure 2. The analysis of the shape of these curves points out two branches: the first branch is due to the interaction between CNTs and the gas, which begins when the gas starts flowing inside the chamber. The second branch is due to the relaxation of the CNTs sensor, which starts when the gas valve is closed. During this second phase, the adsorbed gas is released from the CNTs and pumped out of the chamber.

Figure 2 (Left) shows two sequential responses of the sensor to hydrogen at the same concentration (0.25 ppm), while Figure 2 (Right) presents two measurements carried out at the hydrogen concentrations of 0.02 ppm and 0.16 ppm respectively. Evidently, in the range of pressures analyzed, CNTs respond to $H_{2}$ by increasing their resistivity. Such behavior agrees with other experiments with a similar setup [17,18]. Since the sensibility of the Keithley is much less than the voltage signal fluctuations, the statistical error is directly obtained from the signal oscillation; in the plots proposed in Figure 2 (Left) and (Right) it merely coincides with the thickness of the black line.

The absolute response of the sensor can be appreciated by calculating the responsiveness, which in the present case is defined as:

$$\frac{V\left(t\right)-V_{0}}{V_{0}}$$

where $V_{0}$ is the equilibrium voltage at which the ascent starts. Since the responsiveness does not depend on the dimensions of the system, it is a good physical quantity to characterize the sensor.

**Figure 3:**
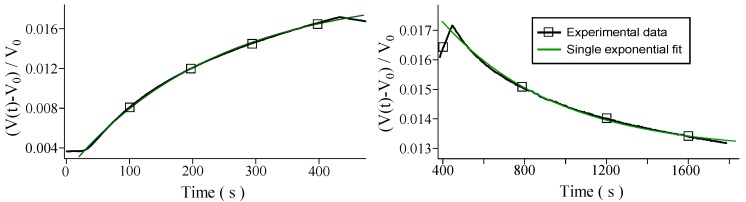
Single exponential fit for the measured curves for hydrogen at 0.16 ppm. (**Left**) Ascent curve; (**Right**) Descent curve.

The responsiveness curves can be analyzed evaluating the characteristic times of the adsorption/desorption process. To this end, data can be fitted with a single exponential function

$$Singlexp\left(t\right)=\left\{\begin{array}{cc} A\left(1-e^{-\frac{t-t_{0}}{\tau }}\right)+B & ascent\\ Ae^{-\frac{t-t_{0}}{\tau }}+B & descent \end{array}\right.$$

using the least-squares method. In Figure 3 the superposition of the fitted model upon the responsiveness data is presented.

**Figure 4:**
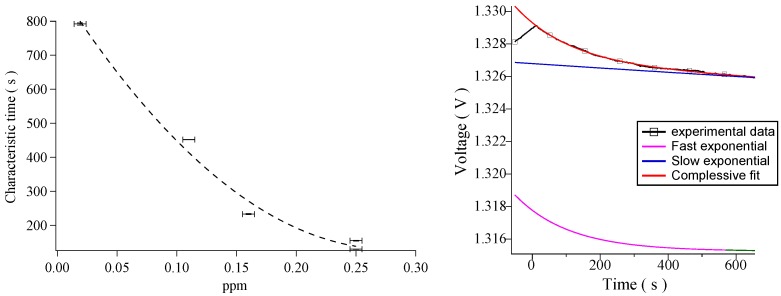
(**Left**) Dispersion plot showing decreasing response times as function of increasing gas concentrations; (**Right**) Decomposition of desorption process in terms of the model used (see text).

From the exponential fit the characteristic adsorption time *τ* can be derived with its statistical error. In Figure 4 (Left) the values of *τ* found for the ascent branches are displayed as a function of the concentration of $H_{2}$. From this plot it is clear that, for the ascents, *τ* decreases as the concentration of gas increases and this is reasonable since the greater the quantity of gas introduced, the more frequent the interaction with the CNTs. Figure 4 (Left) basically represents the characteristic time calibration curve for the sensor.

Although the fitted model results in a reasonable response, a closer look to the fit parameters is mandatory, to obtain quantitative results. The attention is focused on the reduced $\chi^{2}$ values, shown in Table 1a. In most of the cases, both for ascents and descents, the fits must be rejected by virtue of the $\chi^{2}$ test, because the observed significance level is less than 1%. It is important to stress that this means that the collected data are not distributed according to a single exponential function and therefore it is reasonable to make a different choice for the fitting function.

Taking into account the hypothesis of two distinct adsorption processes, which evidently may be characterized by two different proper times $\tau_{1}$ and $\tau_{2}$, the most reasonable choice is to take a linear combination of two exponential functions:

$$Doublexp\left(t\right)=\left\{\begin{array}{cc} \sum_{i=1}^{2}A_{i}\left(1-e^{-\frac{t-t_{0_{i}}}{\tau_{i}}}\right)+B & ascent\\ \sum_{i=1}^{2}A_{i}e^{-\frac{t-t_{0_{i}}}{\tau_{i}}}+B & descent \end{array}\right.$$

**Figure 5:**
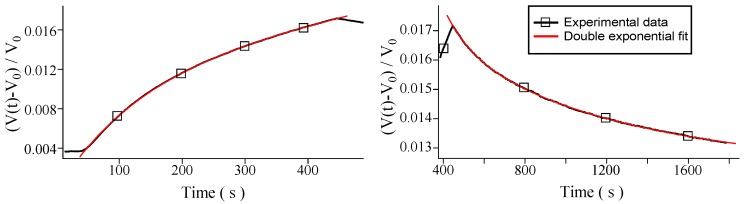
Double exponential fit for hydrogen. As it can be seen, a better agreement is reached with respect to single exponential fit. (**Left**) Ascent curve; (**Right**) Descent curve.

Again, the least-square method is used as fitting procedure. In Figure 5 the doublexp fit is superimposed on the responsiveness data. As before, to evaluate the goodness of the fit, reduced $\chi^{2}$ values are calculated and analyzed in Table 1b. In these cases, all the fits show an acceptable observed significance level and they cannot be rejected. This suggests that two interaction processes take place simultaneously and that both are detectable by the kind of measurements described in this work.

The numerical values for the characteristic times $\tau_{1}$ and $\tau_{2}$ resulting from the doublexp fit are listed in Table 2. Errors for the reported data are of the order of 10% or below.

The descent characteristic times are particularly important because they can carry information about the strength of interaction between the adsorbed gas and the CNTs. In particular, Table 2 highlights that for every descent, a slower process coexists with a faster process. In Figure 4 (right) this peculiar behavior is visually enlightened by plotting the two different exponential contributions separately, under their linear combination. From this plot it can be appreciated that the velocities of the two processes are remarkably different and this is the main reason why both phenomena can be detected. This allows to easily recognize the two phenomena, physi(de)sorption and chemi(de)sorption; the faster process in the descent is associated with physi(de)sorption of $H_{2}$ from the CNTs while the slower process in the descent is associated with chemi(de)sorption of $H_{2}$ from the CNTs defects.

The two measurements at 0.25 ppm highlight the role of the adsorbed $H_{2}$ which does not leave the CNTs after the gas is pumped out of the chamber. Indeed, this permanently chemisorbed $H_{2}$ affects the reactivity of the sensors so that the fitted characteristic times (Table 2) at the same concentration (0.25 ppm) differ a bit from each other.

**Figure 6:**
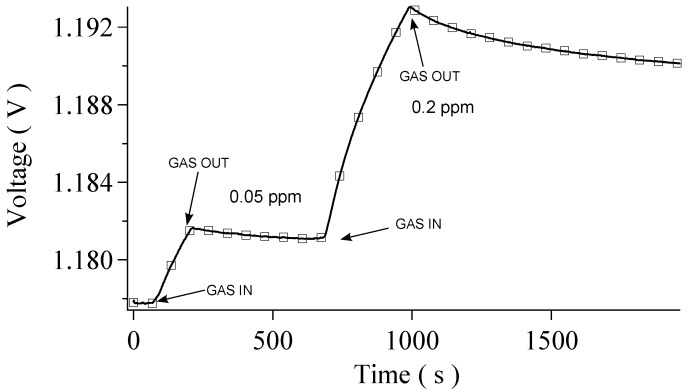
Set of data for the ${\mathrm{CO}}_{2}$ case. Data show the response of the sensor for gas concentrations of 0.05 ppm and 0.20 ppm.

**Figure 7:**
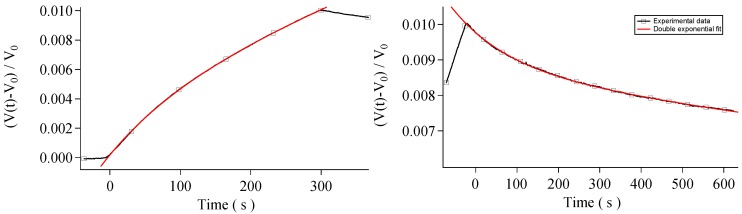
Double exponential fit for carbon dioxide. (**Left**) Ascent curve; (**Right**) Descent curve.

The same analysis of $H_{2}$ has been carried out for ${\mathrm{CO}}_{2}$ (Figure 6). Also, in this case, the interaction between ${\mathrm{CO}}_{2}$ and CNTs leads to an increase in resistivity, as expected from the literature [14]. Measurements have been performed with a ${\mathrm{CO}}_{2}$ concentration of 0.05 ppm and 0.20 ppm. As in the $H_{2}$ case, for ${\mathrm{CO}}_{2}$ single exponential fits are performed, but the analysis of the $\chi^{2}$ values suggests that the fit function is not well-representing the data. In contrast, the double exponential fit (Figure 7) is acceptable by means of $\chi^{2}$ test and the coexistence of two characteristic times is seen also in this case. The descent characteristic times $\tau_{1}$ and $\tau_{2}$ are approximately an order of magnitude different also for ${\mathrm{CO}}_{2}$, that is, a slower process and a faster one are detected separately. As for $H_{2}$, the faster process in the descent is associated with physi(de)sorption while the slower process in the descent is associated with chemi(de)sorption. Data are presented in Table 3.

## 4. Conclusions

It was shown here that it is possible to describe the response of CNTs sensors in terms of a double exponential function. From the data shown in Table 2 the double exponential fit shows the presence of two different phenomena taking place during the desorption process. The different times are linked to physi(de)sorption and chemi(de)sorption, with the slower process due to the chemisorbed adsorbates. The separate detection of the two adsorption processes can be used to improve the selectivity of a CNTs based sensor. In fact, the physisorption time and the chemisorption time could be used as independent fingerprints to recognize the compound to be sensed and this may allow to build better CNTs-based sensors. A further step to completely verify and validate the thesis discussed in this paper is to check the double exponential sensing behavior in a wider range of pressure and using a greater variety of compounds to be sensed, such as electron-donor gases. Furthermore, the approach presented is general and can in principle be transferred to other CNT-based sensors, such as metal nanoparticles-decorated CNT sensors.

## Figures and Tables

**Table 1:** (**a**) Reduced $\chi^{2}$ values for the single fit exponential for $H_{2}$ curves. First row refers to the ascent while the second to the descent part of the curves; (**b**) Reduced $\chi^{2}$ values for the double fit exponential for $H_{2}$ curves. First row refers to the ascent while the second to the descent part of the curves. The values of the reduced $\chi^{2}$ are below the 95th percentile therefore the fitted model cannot be rejected.

(a)					
**Concentration**	**0.02 ppm**	**0.11 ppm**	**0.16 ppm**	**0.25 ppm**	**0.25 ppm**
$\chi^{2}$ ascent	1.64	70.14	92.06	39.46	3.28
$\chi^{2}$ descent	1.38	4.95	28.66	2.69	2.97
**(b)**					
**Concentration**	**0.02 ppm**	**0.11 ppm**	**0.16 ppm**	**0.25 ppm**	**0.25 ppm**
$\chi^{2}$ ascent	1.32	1.16	1.72	1.29	1.16
$\chi^{2}$ descent	1.22	3.21	1.75	0.82	0.66

**Table 2:** Values of the *τ* for both the ascent and descent region for all the sensing cycles performed, as given by the double exponential model.

Characteristic Times as Given by the Model for the $H_{2}$ Case					
**Concentration (ppm)**	**0.02**	**0.11**	**0.16**	**0.25**	**0.25**
$\tau_{1}$ ascent (s)	327	76.7	104	53.5	47.6
$\tau_{2}$ ascent (s)	2060	892	1250	783	511
$\tau_{1}$ descent (s)	254	161	130	63.3	35.2
$\tau_{2}$ descent (s)	8200	8450	896	494	272

**Table 3:** Values of the *τ* for both the ascent and descent region for all the sensing cycles performed, as given by the double exponential model.

Characteristic times for ${\mathrm{CO}}_{2}$		
**Concentration (ppm)**	**0.05**	**0.20**
$\tau_{1}$ ascent (s)	225	96
$\tau_{2}$ ascent (s)	2581	1471
$\tau_{1}$ descent (s)	285	202
$\tau_{2}$ descent (s)	2352	5697
